# Do Magnesium Ions Have Similar Effects as Calcium Ions on Resting Membrane Potential?

**DOI:** 10.3390/membranes16030093

**Published:** 2026-03-02

**Authors:** Anthony Hana, Youngwoo Kim, Joy Bidros, Katie Neglia, Robin L. Cooper

**Affiliations:** 1 Department of Biology, University of Kentucky, Lexington, KY 40506, USA; anthony.hana@uky.edu (A.H.); youngwoo.kim130@topper.wku.edu (Y.K.); joy.bidros@uky.edu (J.B.); kane237@uky.edu (K.N.); 2 The Gatton Academy, 1906 College Heights Blvd. #71031, Bowling Green, KY 42101, USA; 3 Model Laboratory School, 521 Lancaster Ave, Richmond, KY 40475, USA

**Keywords:** K2P, magnesium, membrane potential, NALCN, potassium channel, sodium channel

## Abstract

Maintaining a membrane electrical potential of biological cells is a dynamic process, as some cells have a continually changing potential, like pacemaker cells, while other cells may function with large or small changes in the membrane potential. Additionally, some cells may change their electrical potential when stimulated or inhibited by electrical signals, chemical compounds, or both—either simultaneously or episodically. The persistent leak of K^+^ through two-pore-domain potassium channels (K2P) and of Na^+^ through Na^+^ leak channels (NALCNs) and the action of pumps and exchangers are primarily responsible for maintaining a resting potential. Ca^2+^ ions are known to block the NALCNs and result in a more hyperpolarized membrane potential, with a reduction in Ca^2+^ resulting in a depolarized state. Using the larval muscles of *Drosophila*, the membrane potentials were monitored as Ca^2+^ and Mg^2+^ concentrations were altered. Changes as large as 20 mM of Mg^2+^ had only small effects (1 to 2 mV) on the membrane potential compared to 3–5 mM changes in Ca^2+^ having larger effects (5–10 mV). Although, it appears raised [Mg^2+^] may dampen the changes induced by Ca^2+^. Simulations of the G-H-K equation estimate the changes in permeability of Na+ (pNa). These experiments are significant, as the clinical severity of hypocalcemia and hypercalcemia may also depend on Mg^2+^ levels.

## 1. Introduction

It is well established that the concentration of extracellular free Ca^2+^ [Ca^2+^]_O_ can impact the resting membrane potential of most cell types by blocking the influx of Na^+^ through Na^+^ leak channels (referred to as NALCNs) [1]. The magnitude of the effect of [Ca^2+^]_O_ on the resting membrane potential depends on the density of the NALCN and K2P channels. K2P channels are K^+^ leak channels, also referred to as two-pore-domain potassium channels [2,3,4]. The K2P channels consist of several subtypes. Depending on which subtype is constitutively open or modulated to be open, as based on the pH, stretch, or other environmental factors, there will be variable effects to the alteration of the membrane potential by [Ca^2+^]_O_.

There is generally a dose-dependent effect of [Ca^2+^]_O_ on the NALCN block; thus, increasing [Ca^2+^]_O_ produces a more hyperpolarized resting membrane potential (RP), as the RP is driven towards the E_K_ (equilibrium potential for K^+^), which then limits the effect of any further increase in [Ca^2+^]_O_ on the RP. A lower [Ca^2+^]_O_ allows Na^+^ to leak in and depolarize the RP towards E_Na_. This has been demonstrated in the muscle model of larval *Drosophila* [5,6] as well as many other model systems such as the neurons of blue crabs [7] and GH3 cells of a rat pituitary cell line [8]. The effects of reduced [Ca^2+^]_O_ on membrane excitability in humans account for the neurological changes associated with Chvostek’s and Trousseau’s signs [9,10]. Low [Ca^2+^]_O_ is life threatening to most animals not just due to effects on the nervous system but all cells, such as the cardiac system and skeletal muscles, which depend on Ca^2+^ flux.

The goal of this investigation is based on the concept that free Mg^2+^ generally blocks voltage-gated Ca^2+^ channels. Thus, it may also be possible that free Mg^2+^ can also act like Ca^2+^ in blocking or interacting with the NALCN channel and thus also alters the resting membrane potential. If this is the case, then this may explain one of the mechanisms in the rationale of why hypomagnesemia is associated with epileptic seizures in mammals [11,12]. Just as hypocalcemia can lead to hyperexcitability in neurons, hypomagnesemia may as well. In addition, hypomagnesemia combined with hypocalcemia may produce additive or synergistic effects on membrane excitability. This has not been well addressed previously, but low levels of [Mg^2+^]_O_ and [Ca^2+^]_O_ are known to occur in some pathological states, particularly with kidney dysfunction, and during some therapeutic treatments [13,14,15].

The NALCN protein has been shown to have two alternatively spliced isoforms, with one that is structurally similar to voltage-gated Na+ channels and another which is similar to voltage-gated Ca^2+^ channels. The form that is similar to Na^+^ channels is referred to as EEKE or EKEE and the one resembling Ca^2+^ channels as EEEE [16]. It is interesting that both subtypes are expressed in snails and have been shown to be differentially expressed in differing tissues [16]. Thus, it is possible to be the case for other invertebrates, such as *Drosophila*, as well as mammals. This topic remains to be fully investigated with proteomics in various animal and tissue models. In addition, physiological investigations are warranted to address how [Ca^2+^]_O_ as well as [Mg^2+^]_O_ and other ions may interact with the Na^+^ flux of the NALCN pore.

**Figure 1:**
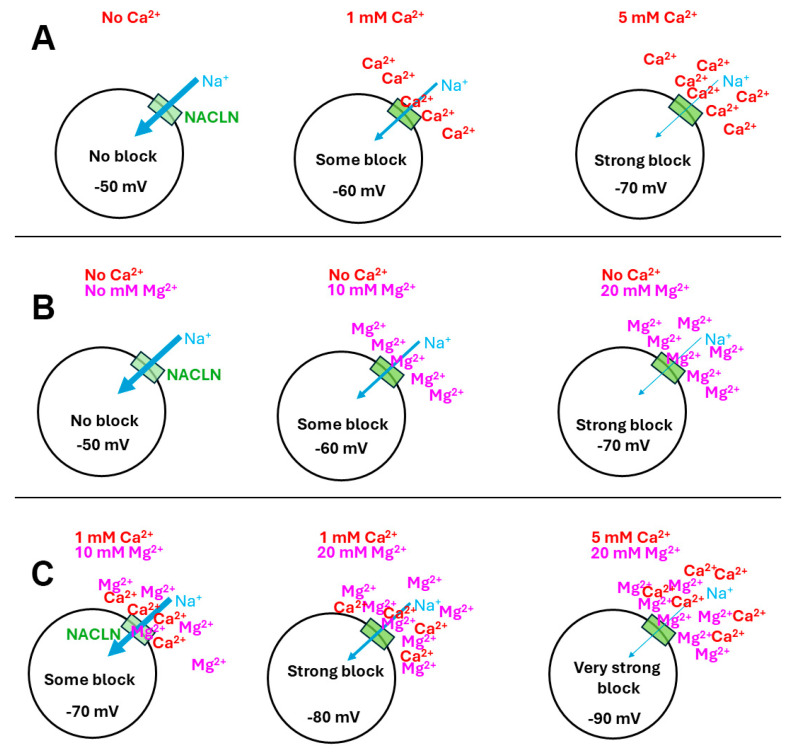
A schematic representation of the effects of extracellular [Ca^2+^]_O_ and [Mg^2+^]_O_ on the resting membrane potential by action on the NALCNs (green box). (**A**) As [Ca^2+^]_O_ is increased, the NALCNs are increasingly blocked, resulting in a more negative membrane potential. (**B**) Without the presence of [Ca^2+^]_O_ and increasing [Mg^2+^]_O_, there is an increasing block on the NALCNs resulting in a more negative membrane potential. (**C**) As both [Ca^2+^]_O_ and [Mg^2+^]_O_ are increased, they are both adding to the blocking of NALCNs; however, the high [Mg^2+^]_O_ may interfere with the action of Ca^2+^ on the NALCNs.

As schematically illustrated in Figure 1A, the increase in [Ca^2+^]_O_ will result in an increased block in the number of NALCNs, thus resulting in the hyperpolarization of the membrane potential. The same logic for [Ca^2+^]_O_ would apply for [Mg^2+^]_O_, blocking NALCNs in the absence of [Ca^2+^]_O_ (Figure 1B). However, with [Ca^2+^]_O_ and [Mg^2+^]_O_ present, there would be competitive competition for blocking the NALCNs and potentially displacing the opposing ion based on concentration and affinity to the NALCN channel (Figure 1C).

### Simulations

In an attempt to estimate the effects of the changes in the Na^+^ permeability (pNa) with changes in the influx through the NALCNs, the Goldman–Hodgkin–Katz (GHK) equation for ion movement and voltage can be generally used. Given that only one parameter (i.e., pNa) is assumed to be changing with the effects on the NALCNs, the other variables in the GHK equation can remain constant. Various parameters in the GHK equation can be used to fit the empirical data; however, by only altering the term of pNa, a general understanding in the degree of change expected can be estimated with simulations on the membrane potential [5,17,18]. Simulations using the GHK equation do not consider the non-linear effects of pumps and exchangers but do provide a measure for the theoretical effect on pNa based on empirical data [19,20].

The model preparation of body wall muscles in larval *Drosophila* provides a readily accessible preparation for measuring membrane potential and the effects of altering [Ca^2+^]_O_ and [Mg^2+^]_O_. In addition, the E_K_ is estimated to be about −90 mV [21,22] of the larval muscle, thus allowing for a range in the RP to change with various manipulations of the [Ca^2+^]_O_ and [Mg^2+^]_O_ from the general RP of ~60 mV with use of the standard saline for larval *Drosophila*. In addition, the concentration of [Ca^2+^]_O_ was previously measured to be around 1.5 mM in the larval hemolymph [23]. The extensive foundational information in the physiology of the *Drosophila* larval muscle provides a basis to further build the physiological knowledge using this model’s organism. The hypothesis being tested in this study is that free Mg^2+^ will behave like free Ca^2+^ in blocking the NALCN, but with a differing affinity, and will show concentration-dependent effects as Ca^2+^.

## 2. Materials and Methods

Early third-instar *Drosophila* CS larvae were used (50–70 h) post-hatching. The larvae were maintained at room temperature, ~21 °C, in vials partially filled with a cornmeal–agar–dextrose–yeast medium. These *Drosophila* strains were obtained from the Bloomington *Drosophila* Stock Center (BDSC, Bloomington, IN, USA). The technique for dissecting larvae and measuring membrane potential was described previously [5]. The segmental nerves were transected close to the larval brain to prevent spontaneous evoked contractions induced from the CNS of the larvae. The dish for the larvae was recorded, and this is illustrated in video format [24].

In brief, the early third-instar larval *D. melanogaster* was dissected in physiological saline. The m6 muscle in segment 2 was used throughout these investigations. To monitor the transmembrane potentials of the body wall muscle (m6) of third-instar larvae, a sharp intracellular electrode (30 to 40 megaohm resistance) filled with 3M KCl impaled the fiber. An Axoclamp 2B (Molecular Devices, Sunnyvale, CA, USA) amplifier and 1 X LU head stage were used. Data were collected using a PowerLab/4sp (ADInstruments, Colorado Springs, CO, USA) and analyzed with LabChart 7.0 (ADInstruments, Colorado Springs, CO, USA), the data were recorded on a computer at a 20 kHz sampling rate, along with the use of a NPI GMbH filter (type EPMS07 DPA 2F, from Adam and List Associate, Ltd., 1100 Shames Drive, Westbury, NY 11590, USA) and low-pass filtered at 3.0 kHz with no high-pass filtering.

Modified basal standard HL3 saline was used for the initial dissection (NaCl 70 mM, KCl 5 mM, MgCl_2_·6H_2_O 20 mM, NaHCO_3_ 10 mM, Trehalose 5 mM, sucrose 115 mM, BES 25 mM, and CaCl_2_·2H_2_O 1 mM, and pH 7.1) [23,25]. The bathing saline was then exchanged for one containing a different concentration of CaCl_2_·2H_2_O and/or MgCl_2_·6H_2_O, as described in the Results. For each saline exposure, the preparations were left to incubate for 3 min before recording the membrane potential. An exception was made for saline with 0 mM [Mg^2+^]_O_ and [Ca^2+^]_O_ due to the motor nerve being hyperexcitable and spontaneously firing, causing muscle contractions. So, this saline make up was only incubated for 1 min prior to recording the membrane potential. The pH of all salines was monitored prior to experiments daily and maintained at 7.2.

The raw values of membrane potential are graphed for each preparation. Paired *t*-test or a two-way ANOVA were used to compare changes in membrane potential for different conditions within a paradigm. Percentage changes in membrane potential were determined from the initial values in normal saline as compared to the different saline used to compare the effects within a group. This is a means to normalize preparations with varying membrane potentials. This was determined by the absolute difference (initial experiment) being divided by the initial value and the resultant value being multiplied by 100 for the percentage change. The percentage change from the initial membrane potential to various media was determined for each paradigm.

Each paradigm with the concentrations of Ca^2+^ and Mg^2+^ were as follows:

Paradigm 1: Constant 1 mM [Ca^2+^], Altered [Mg^2+^]

20 mM [Mg^2+^] → 10 mM [Mg^2+^] → 0 mM [Mg^2+^] → 20 mM [Mg^2+^].

Paradigm 2: Constant 10 mM [Mg^2+^], Altered [Ca^2+^]

1 mM [Ca^2+^] → 0 mM [Ca^2+^] → 1 mM [Ca^2+^].

Paradigm 3: Constant 10 mM [Mg^2+^], Altered [Ca^2+^]

1 mM [Ca^2+^] → 5 mM [Ca^2+^] → 1 mM [Ca^2+^].

Paradigm 4: Constant 20 mM [Mg^2+^], Altered [Ca^2+^]

1 mM [Ca^2+^] → 5 mM [Ca^2+^] → 1 mM [Ca^2+^].

Paradigm 5: Constant 20 mM [Mg^2+^], Altered [Ca^2+^]

1 mM [Ca^2+^] → 0 mM [Ca^2+^] → 1 mM [Ca^2+^].

Paradigm 6: Constant 1 mM [Ca^2+^], Altered [Mg^2+^]

10 mM [Mg^2+^] → 0 mM [Mg^2+^] → 10 mM [Mg^2+^].

Paradigm 7: Constant 5 mM [Ca^2+^], Altered [Mg^2+^]

10 mM [Mg^2+^] → 0 mM [Mg^2+^] → 10 mM [Mg^2+^].

Paradigm 8: Constant 0 mM [Ca^2+^], Altered [Mg^2+^]

10 mM [Mg^2+^] → 0 mM [Mg^2+^] → 10 mM [Mg^2+^].

The general procedure was to examine the effects on membrane potential with an initial condition, changed condition, and then back to the original condition.

The simulations were conducted with a Python-integrated environment (i.e., Python extension of the VSCode platform) and a GitHub code (1 November 2025), which can be downloaded here: https://github.com/ywkim17/Educational_Book/blob/main/GHK.py (accessed on 1 November 2025). The code is named “GHK.py”

The code named “Nernst.py” was used via VSCode software, as shown in Appendix A and in Elliott and Cooper [5].

The initial values for starting the simulations for the larval *Drosophila* muscle were as shown below.

[K^+^]_i_ = 190 mM (estimated for *Drosophila* muscle);

[K^+^]_O_ = 5 mM (saline);

[Na^+^]_i_ = 12 mM (assumed for *Drosophila* muscle);

[Na^+^]_O_ = 80 mM (saline);

pK = 1 (assumed for muscle);

pNa = 0.001 (starting value assumed for muscle; this parameter is the only one that is altered in the simulations).

## 3. Results

**Figure 2:**
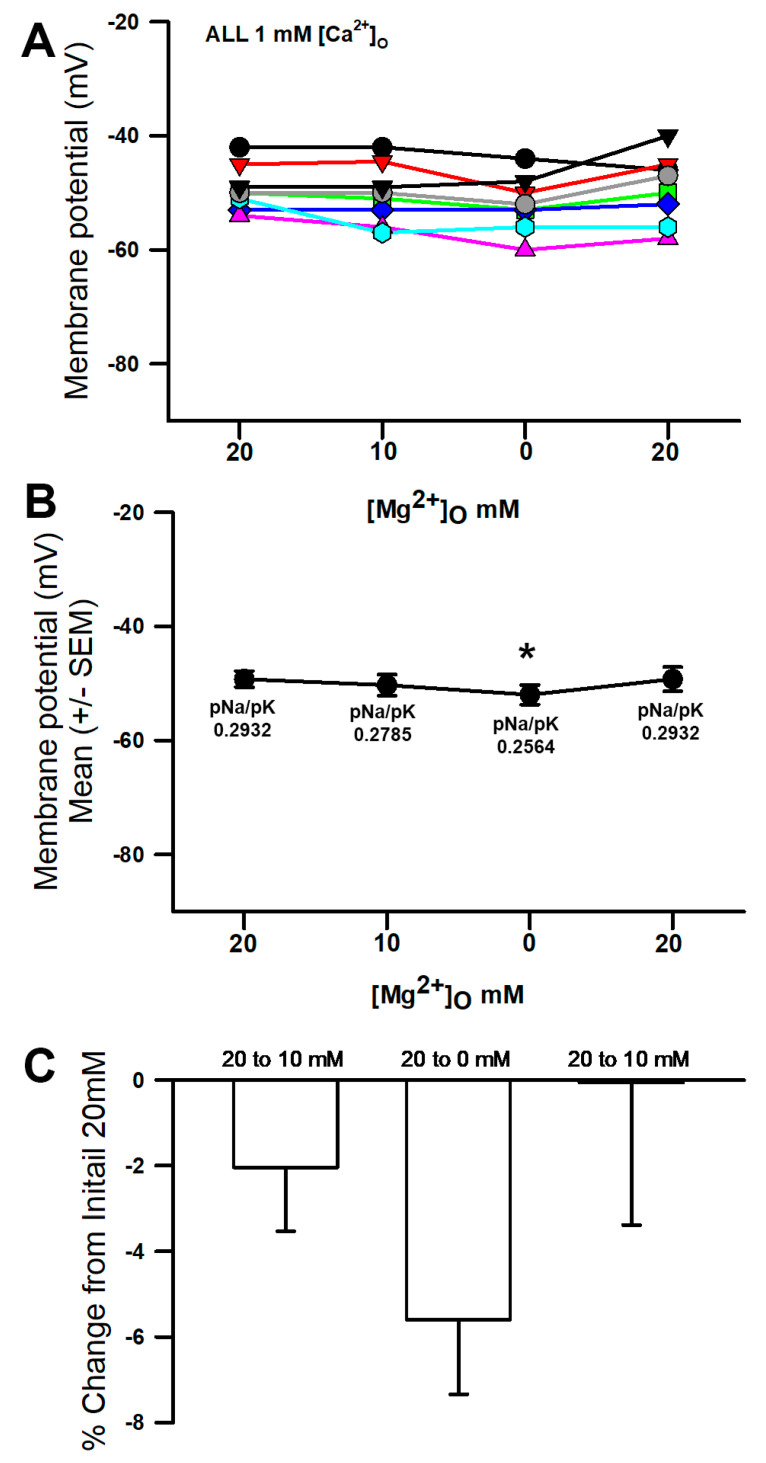
The effects of altering the [Mg^2+^]_O_ on the membrane potential. (**A**) As the [Mg^2+^]_O_ was reduced from 20 mM to 0 mM, the membrane potential became more negative (* *p* < 0.05, paired *t*-test, and N = 8). There was not a significant effect on the membrane potential when changing from 20 to 10 mM [Mg^2+^]_O_. (**B**) The mean (+/−SEM) of the membrane potentials for the individual preparations. (**C**) Since each individual preparation had differing initial membrane potentials, a percent change from the initial was performed. Note the simulated values for pNa/pK in the initial 20 mM and 0 mM [Mg^2+^]_O_ conditions. The different colored traces represent individual preparations.

A variety of salines were used to examine the effects altering [Mg^2+^]_O_ on the membrane potential along with changes in the [Ca^2+^]_O_. The membrane of the muscle hyperpolarized when exposed to a reduced concentration of Mg^2+^ in the saline from 20 mM to 0 (Figure 2A; * *p* < 0.05, paired *t*-test, and N = 8). The average of the preparations reveals the overall trend (Figure 2B). There were only slight decreases in the values from 20 to 10 mM [Mg^2+^]_O_ and 10 to 0 mM [Mg^2+^]_O_, which were then demonstrated to not be significant at *p* < 0.05 for a paired *t*-test. Since each preparation varied in the initial values, a percentage change from the initial values was obtained to normalize the differences. The averaged percentage change was small for the saline containing Ca^2+^ (Figure 2C). The simulated change in the pNa to best match the membrane potential for 20 mM to 0 mM [Mg^2+^]_O_ was 0.2932 to 0.2564, which would allow for the hyperpolarization of the membrane. The parameters for the initial conditions were pK = 1 and pNa = 0.2932, which would result in a change in the pNa/pK ratio of 0.0368.

**Figure 3:**
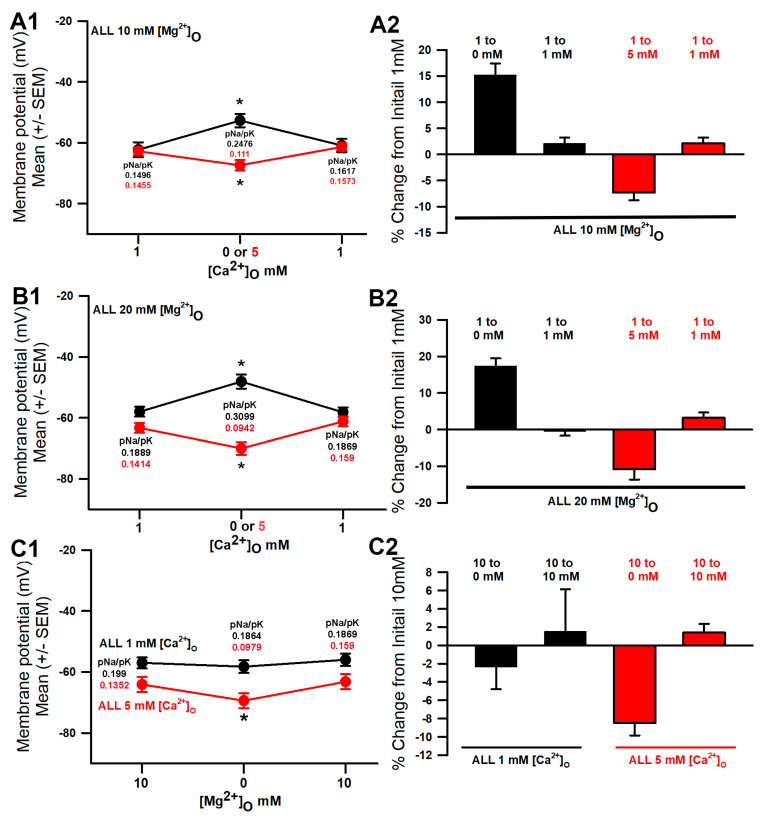
The effects of altering the [Ca^2+^]_O_ on the membrane potential, with [Mg^2+^]_O_ maintained at 10 mM. (**A1**) As the [Ca^2+^]_O_ was reduced from 1 mM to 0 mM, the membrane potential became more depolarized (* *p* < 0.05, paired *t*-test, and N = 10), and when the [Ca^2+^]_O_ was increased from 1 mM to 5 mM, the membrane potential became more negative (* *p* < 0.05, paired *t*-test, and N = 10). (**A2**) Since each individual preparation had differing initial membrane potentials, a percentage change from the initial was performed. (**B1**) The effects of altering the [Ca^2+^]_O_ on the membrane potential, with [Mg^2+^]_O_ maintained at 20 mM. (**A1**) As the [Ca^2+^]_O_ was reduced from 1 mM to 0 mM, the membrane potential became more depolarized (* *p* < 0.05, paired *t*-test, and N = 10), and when the [Ca^2+^]_O_ was increased from 1 mM to 5 mM, the membrane potential became more negative (* *p* < 0.05, paired *t*-test, and N = 10). (**B2**) Since each individual preparation had differing initial membrane potentials, a percentage change from the initial was performed. (**C1**) The effects of altering the [Mg^2+^]_O_ on the membrane potential, with [Ca^2+^]_O_ maintained at 1 or 5 mM. As the [Mg^2+^]_O_ was reduced from 10 mM to 0 mM in the presence of 5 mM [Ca^2+^]_O_, the membrane potential became more hyperpolarized (* *p* < 0.05, paired *t*-test, and N = 10), but not with [Ca^2+^]_O_ maintained at 1 mM. (**C2**) Since each individual preparation had differing initial membrane potentials, a percentage change from the initial was performed for each protocol maintaining [Ca^2+^]_O_ at 1 or 5 mM. Note the simulated values for pNa in each condition.

In saline containing 10 mM [Mg^2+^]_O_ and altering the [Ca^2+^]_O_, there were significant changes, either increasing the [Ca^2+^]_O_ from 1 to 5 mM or decreasing the [Ca^2+^]_O_ from 1 to 0 mM (Figure 3(A1); *p* < 0.05, paired *t*-test, and N = 10). Upon returning the saline back to the original concentration of 1 mM [Ca^2+^]_O_, the membrane potential recovered well. With the reduced [Ca^2+^]_O_, the pNa increased from 0.1496 to 0.2476 in the GHK simulation to best match the membrane potential. The increase in [Ca^2+^]_O_ to 5 mM resulted in an estimated pNa decreasing to 0.111. The change in the pNa/pK ratio would then indicate one of 0.098 for 0 mM and 0.0386 for 5 mM [Ca^2+^]_O_. The percentage changes from the initial values were obtained to normalize the differences. The averaged percentage changes were larger for alteration in the [Ca^2+^]_O_ as compared to [Mg^2+^]_O_, with [Ca^2+^]_O_ being maintained at 1 mM (Figure 2C compared to Figure 3(A2)).

When the [Mg^2+^]_O_ was maintained at 20 mM, and the [Ca^2+^]_O_ was increased to 5 or decreased to 0 mM, the membrane potential also significantly changed (Figure 3(B1); * *p* < 0.05, paired *t*-test, and N = 10). With a short exposure of 3 min to the low or high concentration of [Ca^2+^]_O_ and a return back to the original concentration of 1 mM [Ca^2+^]_O_, the membrane potential recovered well. For the simulated changes in pNa for the decrease in [Ca^2+^]_O_, the pNa increased from 0.1889 to 0.3099 to obtain the best match in the membrane potential. The increase in [Ca^2+^]_O_ to 5 mM resulted in an estimated decrease in pNa to 0.0942. The change in the pNa/pK ratio would then indicate one of 0.121 for 0 mM and 0.0947 for 5 mM [Ca^2+^]_O_. As for the 10 mM [Mg^2+^]_O_, the percentage changes from the initial values were obtained to normalize the differences. The averaged percentage changes were larger for the alteration in [Ca^2+^]_O_ when [Mg^2+^]_O_ was maintained at 20 mM as compared to being maintained at 10 mM (Figure 3(B2) compared to Figure 3(A2)).

In the presence of constant [Ca^2+^]_O_ of 1 mM or 5 mM and then changing [Mg^2+^]_O_ from 10 mM to 0 mM, the membrane potential significantly changed, but only for the condition of the 5 mM [Ca^2+^]_O_ (Figure 3(C1), * *p* < 0.05, paired *t*-test, and N = 10). The initial membrane potential was more negative for [Ca^2+^]_O_ at 5 mM than for that of 1 mM, as more NALCNs were blocked and the potential was driven toward E_K_. When reducing [Mg^2+^]_O_ from 10 to 0 mM, an even lesser degree of NALCN blocks occurred, resulting in further hyperpolarization. Upon returning the [Mg^2+^]_O_ back to 10 mM, the membrane potentials were able to recover close to the initial values. Estimating the changes in pNa for the increase in [Ca^2+^]_O_ from 1 mM to 5 mM, the pNa decreased from 0.199 to 0.1352 to obtain the best match in the membrane potential. The change in the pNa/pK ratio would then indicate a change of 0.199 to 0.1864 for 1 mM [Ca^2+^]_O_ and 0.1352 to 0.079 for 5 mM [Ca^2+^]_O_, which resulted in a larger change. As for the previous conditions, a percentage change from the initial values were obtained to normalize the differences. The averaged percent changes were larger for the alteration when [Mg^2+^]_O_ was changed from 10 mM to 0 mM for the constant 5 mM [Ca^2+^]_O_ (Figure 3(C2)).

**Figure 4:**
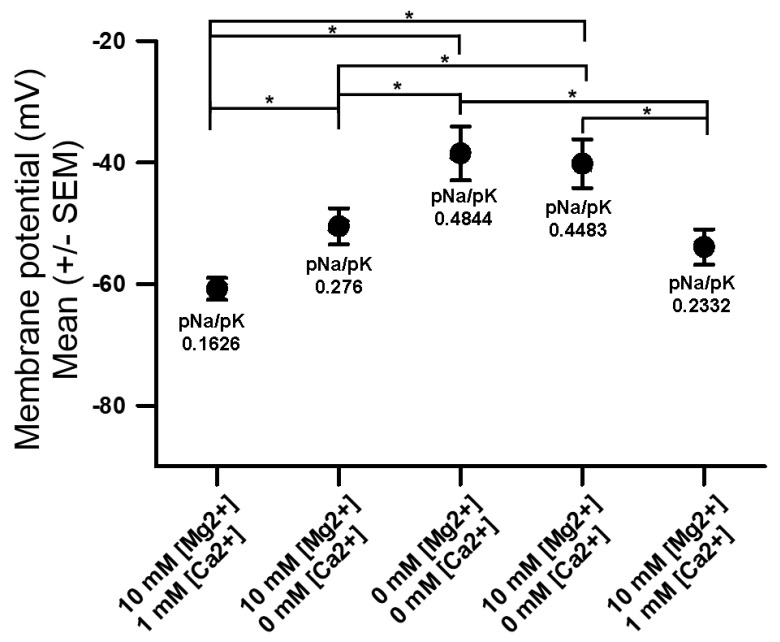
The effects of altering [Ca^2+^]_O_ and [Mg^2+^]_O_ in various cocktails on the membrane potential. As the [Ca^2+^]_O_ and [Mg^2+^]_O_ were reduced together to 0 mM, the membrane potential became more depolarized, and as both ions were added back, the membrane potential became more hyperpolarized (* *p* < 0.05, two-way ANOVA, and N = 10). Note the simulated values for pNa in each condition.

When both the [Ca^2+^]_O_ and the [Mg^2+^]_O_ were decreased to 0 mM, the membrane significantly depolarized, and even when [Ca^2+^]_O_ was at 0 mM and [Mg^2+^]_O_ decreased from 10 to 0 mM, the membrane depolarized substantially (Figure 4 (* *p* < 0.05, two-way ANOVA, and N = 10)). The exposure of 0 mM [Ca^2+^]_O_ and [Mg^2+^]_O_ to 10 mM [Mg^2+^]_O_ with 0 mM [Ca^2+^]_O_ the potential was reversed, as the previous exposure did not allow for the recovery of the membrane potential, even after 3 min of incubation. This is likely due to the spontaneous activity of the motor neuron resulting in larger evoked postsynaptic potentials and muscle contractions, which may result in some membrane damage while monitoring with the intracellular electrode. However, the expected trend in repolarizing the membrane did occur.

Assuming that the only changes occurring in the permeability are due to the NALCN, the estimated values for pNa are shown on Figure 4 for each condition to obtain the best fits for the associated membrane values.

**Figure 5:**
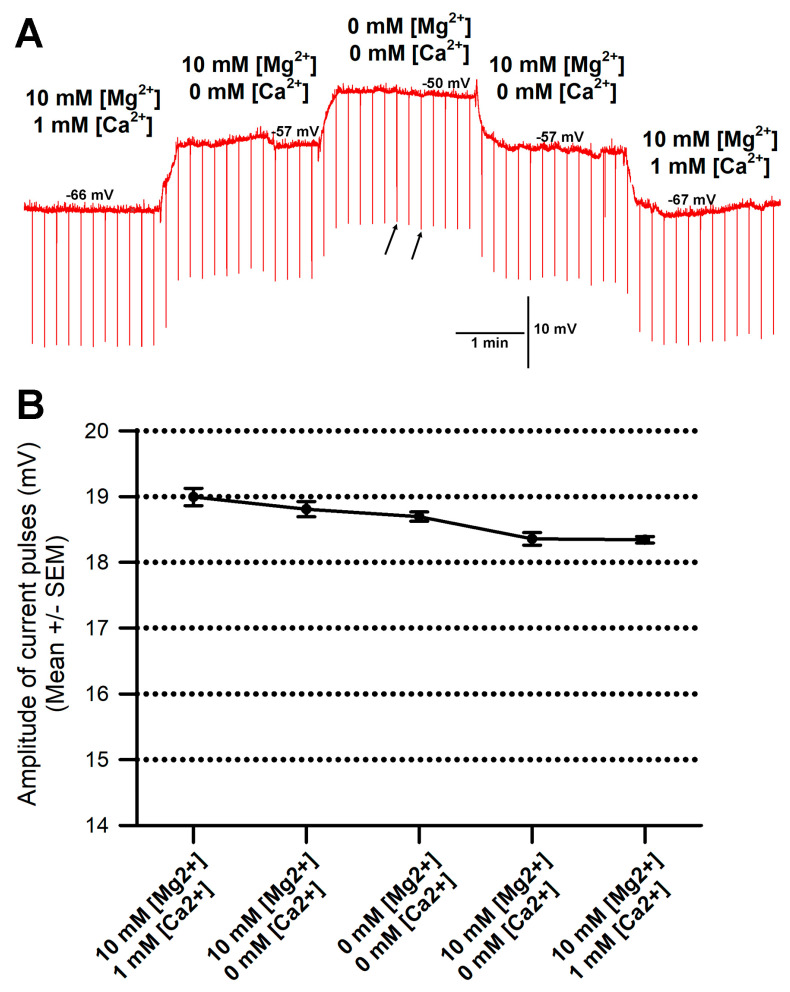
Assay for changes in input resistance of the membrane throughout saline exchanges and particularly for saline with 0 mM [Mg^2+^] and 0 mM [Ca^2+^]. (**A**) A representative trial in which current injections were provided while examining the effect on membrane potential in various salines. Current injection of −2 nA of 100 msec duration every 10 s yielded downward deflections to examine if the membrane resistance was compromised in the various salines. The two small arrows indicate the fluctuations in the amplitudes for a given saline. Acquisition rate was 20 KHz, and the largest deflection within six current pulses was used to obtain an average amplitude, as shown in B. (**B**) The mean amplitude of the current injections shown in A. Note the change is less than 1 mV throughout the entire trial of exchanging saline bathing media.

There was concern that the membrane resistance might have been compromised due to the exposure of low concentrations of Mg^2+^ and Ca^2+^ and the membrane being depolarized. However, when exchanging the saline back to higher concentrations of Mg^2+^ and Ca^2+^, the membrane potential would trend back to the initial values of more negative potentials. Thus, the cell membrane was not severely damaged, even in the cases of slight twitching during the exposure to 0 mM [Mg^2+^] and 0 mM [Ca^2+^]. To examine the extent that the membrane might have been slightly damaged during the saline exchanges and muscle movements, current pulses were injected (−2 nA of 100 msec duration, every 10 s), and the average amplitude of the responses was measured for six independent preparations, as shown in Figure 5A. The change in the amplitudes of the current pulses was small. The largest change was less than 5 mV within the six preparations when exchanging saline from 10 mM [Mg^2+^] and 1 mM [Ca^2+^] to 0 mM [Mg^2+^] and 0 mM [Ca^2+^]. For the representative trial in Figure 5A, the amplitude of the current pulses is shown in Figure 5B. With a high acquisition rate of 20 KHz and 100 msec pulses, the peak values were reliably measured, but still, there were some variations, as shown by the two arrows in Figure 5A, depicting the changes in the current amplitudes while exposed to a given saline.

**Figure 6:**
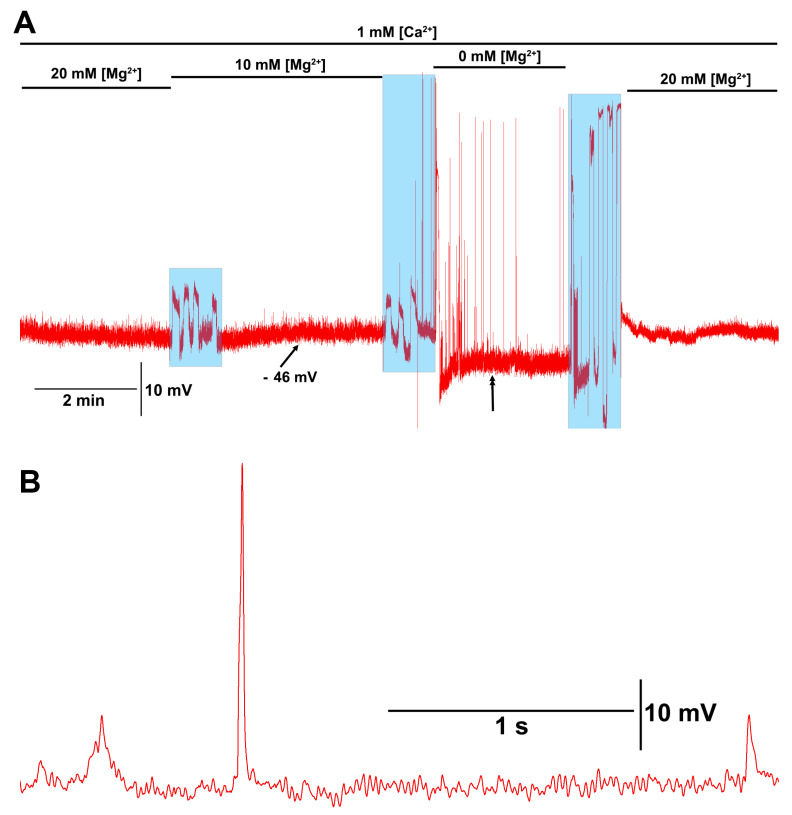
Motor nerve excitation with low [Mg^2+^]_O_. (**A**) When reducing the [Mg^2+^]_O_ from 20 to 10 and finally to 0 mM in the presence of 1 mM [Ca^2+^]_O_, the motor nerve would spontaneously depolarize, resulting in evoked synaptic transmission producing excitatory junction potentials (EJPs). The spontaneous motor nerve firing stopped after changing the bathing saline back to a [Mg^2+^]_O_ of 20 mM. (**B**) An enlarged segment of the trace is shown in (**A**) where the double arrowhead is. Note the large evoked EJP and the quantal events in the trace. The blue transparent boxes is when the saline was exchanged.

When the [Mg^2+^]_O_ was reduced to 0 mM with 1 mM [Ca^2+^]_O_, or when both were reduced to 0 mM, the motor nerve would depolarize enough to induce evoked-like postsynaptic excitatory junction potentials (Figure 6A). These large postsynaptic potentials were larger than single quantal events by 3 to 10 times (Figure 6A,B). When [Ca^2+^]_O_ was 1 mM or greater, along with [Mg^2+^]_O_ at 10 or 20 mM, these larger spontaneous EJPs were not observed.

## 4. Discussion

Magnesium is recognized as an essential mineral for mammalian life, since it serves as a co-factor for many (>300) enzymatic processes, which impact physiological processes associated with glucose regulation and function of the nervous, cardiovascular, as well as muscular systems [26,27,28,29]. The processes impacted relate to cellular dysfunction of the tissues. For humans, a normal range of magnesium in the blood serum level is between 0.75 and 0.95 millimoles (mmol)/L [26,30]. Normal concentrations in the hemolymphs of insects and crustaceans have not been well established, which is likely due to their highly varied environmental conditions and changing developmental stages (i.e., molting and ecdysis) impacting the concentrations. However, the saline designed to mimic the salts within the larval *Drosophila melanogaster* hemolymph (i.e., HL3) provides a sufficient concentration of ionic salts related to Mg^2+^ and Ca^2+^ to provide relatively long physiological recordings for an hour or more [23,25]. Feng et al. [31] showed that a modified HL3 saline, containing Mg^2+^ around 10 to 4 mM from the original 20 mM, provided an even more stable response of the evoked transmission obtained at the larval neuromuscular junction. It was also demonstrated that reducing the Mg^2+^ from 20 mM to 10 or 4 mM in the saline maintained the heart beat for in situ preparations of larval *Drosophila* for longer periods with reduced variability, even with heat stress [32]. A few physiological salines, such as the modified Van Harreveld’s saline [33], maintain the physiological function of neurons and muscles for several hours in crayfish. The modified saline only contains 2.45 mM Mg^2+^ but higher Ca^2+^ at 13.5 mM [34]. In humans, serum magnesium level less than 0.75 mmol/L are considered to be a state of hypomagnesemia [35]. Such low levels are known to result in seizures [11,12] and other physiological disorders [36,37].

The acute cellular mechanisms of the effects of low Mg^2+^ levels have not been well addressed. In this investigation, it was demonstrated that low levels of bathing saline in the *Drosophila* model can have a large impact on the membrane potential, even in the presence of normal Ca^2+^ levels. However, when both Mg^2+^ and Ca^2+^ are very low, the membrane potential of the muscle significantly depolarizes and appears to even allow the motor nerve to spontaneously depolarize, evoking synaptic transmission. The varied pathological conditions in mammals when both Mg^2+^ and Ca^2+^ can be low in the serum, such as for kidney disorders or poor nutrition, can lead to a life-threatening situation. The mechanisms behind the acute need for the balance of these ions may not be that of serving as the co-factors of enzymes or helping to maintain blood glucose levels but that of maintaining a physiological range in the membrane potential of cells.

The effect on the membrane potential for the larval muscles in *Drosophila* with lower [Ca^2+^]_O_ can be offset by higher levels of [Mg^2+^]_O_, and the reverse is also the case for low levels of [Mg^2+^]_O_ being offset by a larger concentration of [Ca^2+^]_O_. These conditions of the membrane potential are likely associated with interactions in the NALCNs. The reciprocal compensation is not the case for the maintenance of evoked synaptic transmission, as Ca^2+^ is necessary to induce vesicular fusion presynaptically, and Mg^2+^ blocks the presynaptic voltage-gated Ca^2+^ channels. Considering that low levels of [Mg^2+^]_O_ and [Ca^2+^]_O_ lead to nerves inducing evoked responses, this suggests how sensitive the nerve is to the reduced level. Unfortunately, the neurons are too small to obtain intracellular recordings from the axons for direct measures. When Ca^2+^ is present, the NALCN on the muscle is sensing some blocks. If Ca^2+^ is raised, more blocking occurs, and the membrane hyperpolarizes. However, if Mg^2+^ is present, there is some competition for the blocking of NALCN by Ca^2+^. Thus, if [Ca^2+^] _O_ stays constant (i.e., 1 mM) and [Mg^2+^]o is reduced, there is less interference from Mg^2+^ interfering with the action of Ca^2+^ in blocking NALCNs, so the effect of Ca^2+^ is greater, and the block becomes stronger. Thus, the membrane hyperpolarizes, with 1 mM [Ca^2+^] _O_ and 10 mM [Mg^2+^] _O_ being changed to 1 mM [Ca^2+^] _O_ and 0 mM [Mg^2+^] _O_.

However, if there is zero [Ca^2+^]_O_, Mg^2+^ now behaves more like Ca^2+^ alone. Higher [Mg^2+^]_O_ blocks the NALCN, and the membrane hyperpolarizes (Figure 3). And lower Mg^2+^]_O_ reduces the block of the NALCN, and the membrane depolarizes. This only occurs without Ca^2+^ present.

**Figure 7:**
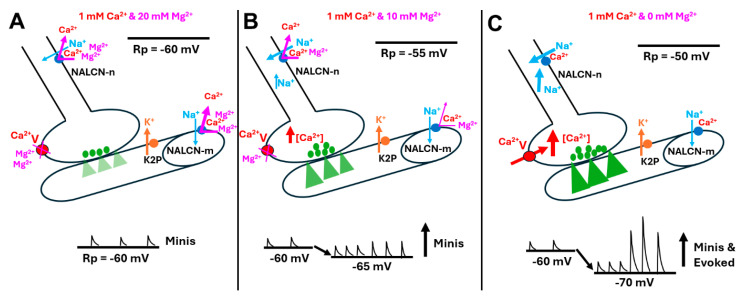
Schematic overview of the potential interactions of [Mg^2+^]_o_ and [Ca^2+^]_o_ on the resting membrane potential of the motor nerve and muscle influencing synaptic activity. (**A**) The standard physiological saline HL3 contains 20 mM [Mg^2+^]_o_ and 1 mM [Ca^2+^]_o_. (**B**) Muscle with the modified HL3 saline trends in a hyperpolarizing direction but not significantly. In both A and B, there is likely some interaction of Mg^2+^ in blocking a fraction of the voltage-gating presynaptic calcium channels (Ca^2+^_V_). There is likely more interaction in the NALCNs on the nerve and muscle, with Mg^2+^ potentially displacing Ca^2+^ from blocking the channel. The trends suggest that Mg^2+^ may also block NALCNs but not as efficiently as Ca^2+^. (**C**) Removing the [Mg^2+^]_o_ and retaining some [Ca^2+^]_o_ in the saline can result in the nerve terminal depolarizing, leading to large excitatory junction potentials, which are similar to nerve evoked responses. Thus, it appears the nerve becomes spontaneously active in low [Mg^2+^]_o_. The muscle fiber hyperpolarizes, adding to the increased driving force of Na^+^ ions through the ionotropic glutamate receptors. The green triangles in the muscle fibers represent the ionic flow through the ionotropic glutamate receptors. All the other colors are representative of the corresponding color of items in the figure.

Thus, the subtypes of NALCN (i.e., EKEE and EEEE), or different isoforms of NALCN, may have varied ratios of expression in the neurons and muscle, as noted as occurring in various tissues in the land snail [16]. This is schematically illustrated in Figure 7, which depicts a neuronal subtype of NALCN-n or a ratio and a muscle subtype of NALCN-m or a ratio.

A standard of clinical care for life-threatening hyperkalemia, which results in the depolarization of membrane potential, is an intravenous injection of calcium gluconate, which has been recommended since the 1960s [38]. The rationale for such treatment began with the initial observations that calcium was important for maintaining the contractions of a frog’s heart in 1883 [39] and that calcium can combat hyperkalemia [40]. Since cardiac abnormalities are one of the first concerns with clinical hyperkalemia, emphasis has been placed on the mechanisms of how calcium can combat the depolarizing effects of hyperkalemia. It is generally assumed that calcium stabilizes the membrane potential and helps to counteract the depolarizing effect. However, a recent study indicates that a higher [Ca^2+^]_O_ does not appear to normalize the effects of hyperkalemia on membrane potential but rather regains electrical conduction through Ca^2+^-dependent propagation in a canine cardiac model [41]. There are multifaceted aspects to consider when addressing effects on mammalian cardiac function, which requires further experimentation. The effect changes in [Ca^2+^]_O_ on the various cell types, from the pacing cells, the transient cells in the AV node, the contracting myocytes in the atria and ventricles, as well as vagal nerve activity on the background chronotropic suppression, have yet to be investigated. In addition, the effects of Ca^2+^ and Mg^2+^ on the NALCNs in the various cell types in mammalian cardiac tissues have yet to be fully investigated. Thus, the results of this study on the effects of Ca^2+^ and Mg^2+^ on membrane potential serve as a foundation for the potential effects in various cell types in many organisms which express NALCNs [1,42]; but apparently, NALCNs are not expressed in plants [43]. There is also a similar homolog (CCH1) to NALCN and its subunit (i.e., Mid1), known as *Saccharomyces cerevisiae* [43,44], which needs to be examined for the effects of Mg^2+^ on its function.

It would be advantageous to know if the *Drosophila* genome has the ability to express various forms of the NALCN channel or if alternative splicing occurs to provide different isoforms. In addition, there could even be varied accessory proteins, such as Mid1 (i.e., “mating induced death” phenotype), also known as Nif-1, which were shown to alter the function of NALCNs in fungi and animals [1,45,46,47]. Thus, identifying the accessory protein for the NALCNs in their expression level is equally as important as the NALCN to fully understand the mechanism behind how the extracellular concentrations of Mg^2+^ and/or Ca^2+^ influence membrane potential. Could it be that those changes in the ionic strength of saline itself had an effect on the membrane potential? Earlier experiments with CoCl_2_ at 1 mM [48] and other experiments with Fe^3+^ (ferric ammonium citrate) at 5 mM [49] were examined, with a negligible effect on the RP (~1%). Thus, it seems unlikely that the ionic strength changes used in this study can account for the membrane potential changes. A model GUV membrane reconstituted with NALCN and examining the effects of Mg^2+^ and/or Ca^2+^ on the channel would be ideal for a laboratory equipped to perform such investigations.

## Data Availability

The original contributions presented in this study are included in the article. Further inquiries can be directed to the corresponding author.

## Appendix A

The parameters used for coding the GHK estimations.
For running the GHK equation to estimate equilibrium potentials, the GitHub code was used:
https://github.com/ywkim17/Educational_Book/blob/main/GHK.py (accessed on 1 November 2025). The code named “GHK.py” was run on the VSCode software.
Import math:
def ghk(R, T, F, P_K, K_in, K_out, P_Na, Na_in, Na_out):
&quot;&quot;&quot;
Compute membrane potential (Vm) in millivolts using the Goldman–Hodgkin–Katz equation.
Parameters:
R: float—Universal gas constant (J/(mol·K))
T: float—Temperature in Kelvin (K)
F: float—Faraday's constant (C/mol)
P_K: float—Permeability of potassium
K_in: float—Intracellular potassium concentration (mM)
K_out: float—Extracellular potassium concentration (mM)
P_Na: float—Permeability of sodium
Na_in: float—Intracellular sodium concentration (mM)
Na_out: float—Extracellular sodium concentration (mM)
Returns:
Vm (float): Membrane potential in millivolts (mV)
&quot;&quot;&quot;
# Calculate RT/F in millivolts
V_T = (R * T)/F * 1000 # Convert to mV
numerator = (P_K * K_out + P_Na * Na_out)
denominator = (P_K * K_in + P_Na * Na_in)
if denominator == 0:
raise ValueError(&quot;Denominator is zero; check input values.&quot;)
Vm = V_T * math.log(numerator/denominator)
return Vm
# Parameter Inputs
R = 8.314 #Constant Value
T = 294.15
F = 96,485 #Constant Value
P_K, K_in, K_out = 1.3, 140, 220
P_Na, Na_in, Na_out = 0.001, 15, 10
# Compute Membrane Potential
Vm = ghk(R, T, F, P_K, K_in, K_out, P_Na, Na_in, Na_out)
print(f&quot;\nMembrane potential: {Vm:.2f} mV&quot;)
